# Crystal structure and Hirshfeld surface analysis of 2-hy­droxy-7-meth­oxy-1,8-bis­(2,4,6-tri­chloro­benzo­yl)naphthalene

**DOI:** 10.1107/S2056989019012118

**Published:** 2019-09-10

**Authors:** Toyokazu Muto, Kikuko Iida, Keiichi Noguchi, Noriyuki Yonezawa, Akiko Okamoto

**Affiliations:** a Department of Organic and Polymer Materials Chemistry, Tokyo University of Agriculture and Technology, Koganei, Tokyo 184-8588, Japan; b Instrumentation Analysis Center, Tokyo University of Agriculture and Technology, Koganei, Tokyo 184-8588, Japan

**Keywords:** crystal structure, non-coplanar accumulated aromatic rings structure, twisted aroyl group, intra­molecular O—H⋯O hydrogen bond, short contacts involving chloro group, herringbone pattern, Hirshfeld surface analysis, two-dimensional fingerprint plots

## Abstract

In the title compound, C_25_H_12_Cl_6_O_4_, the two carbonyl groups are oriented in a same direction with respect to the naphthalene ring system and are situated roughly parallel to each other, while the two 2,4,6-tri­chloro­benzene rings are orientated in opposite directions with respect to the naphthalene ring system: the carbonyl C—(C=O)—C planes subtend dihedral angles of 45.54 (15) and 30.02 (15)° to the naphthalene ring system are. The dihedral angles formed by the carbonyl groups and the benzene rings show larger differences, the C=O vectors being inclined to the benzene rings by 46.39 (16) and 79.78 (16)°. An intra­molecular O—H⋯O=C hydrogen bond forms an *S*(6) ring motif. In the crystal, no effective inter­molecular hydrogen bonds are found; instead, O⋯Cl and C⋯Cl close contacts are observed along the 2_1_ helical-axis direction. The Hirshfeld surface analysis reveals several weak interactions, the major contributor being Cl⋯H/H⋯Cl contacts.

## Abstract 2

The title compound has a non-coplanar accumulated aromatic rings structure. In the mol­ecule, the two carbonyl groups are oriented in the same direction with respect to the naphthalene ring system and are situated roughly parallel to each other, whereas the two 2,4,6-tri­chloro­benzene rings are orientated in opposite directions with respect to the naphthalene ring system.

## Chemical context   

*o*-Hydroxyaryl ketones are generally recognized to be important precursors in the preparation of valuable products such as drugs, cosmetics, dyes and pesticides (Choy & Kwong, 2013 ▸; Naeimi *et al.*, 2014 ▸; Nimnual *et al.*, 2015 ▸). The preparation methods reported include, for example, Fries rearrangement of phenolic esters (Murashige *et al.*, 2011 ▸), acyl­ation of benzo­quinone and derivatives (Schiel *et al.*, 2001 ▸), coupling reactions of nitriles with boronic acids (Zhou & Larock, 2004 ▸), direct C—H bond aryl­ation of 2-hy­droxy­benzaldehydes (Lee & Yi, 2015 ▸; Weng *et al.*, 2010 ▸), and microwave-assisted direct benzoyl­ation of phenols under solvent-free or ionic liquid conditions (Tran *et al.*, 2017 ▸). The neighbouring carbonyl and hy­droxy groups contribute to the regio- and chemoselectivities in these reactions. Conformational studies of hydroxyaryl ketones in the solid state and in solution have attracted considerable inter­est (Siskos *et al.*, 2015 ▸; Nonhebel, 1968 ▸). Since the discovery of an effective method for diaroylation at the 1,8(*peri*)-positions of the naphthalene ring core and the related reactions (Okamoto & Yonezawa, 2009 ▸; Okamoto *et al.*, 2011 ▸; Okamoto, Mitsui *et al.*, 2012 ▸), we have reported on the spatial organization of 1,8-diaroylated naphthalenes and homologous compounds in both the solid state and solution (Okamoto, Watanabe *et al.*, 2012 ▸; Yoshiwaka *et al.*, 2015 ▸; Okamoto *et al.*, 2015 ▸; Ohisa *et al.*, 2018 ▸). In the crystal structures of these compounds, which have non-coplanar accumulated aromatic rings, mol­ecules are arranged by weak inter­molecular inter­actions, such as non-classical hydrogen bonds and van der Waals inter­actions. Thus, the accumulation structures of 1,8-diaroylated naphthalenes are drastically changed by simple mol­ecular modifications. Herein, we report on the crystal structure and Hirshfeld surface analysis of the title hydroxyaryl ketone, 2-hy­droxy-7-meth­oxy-1,8-bis­(2,4,6-tri­chloro­benzo­yl)naphthalene.
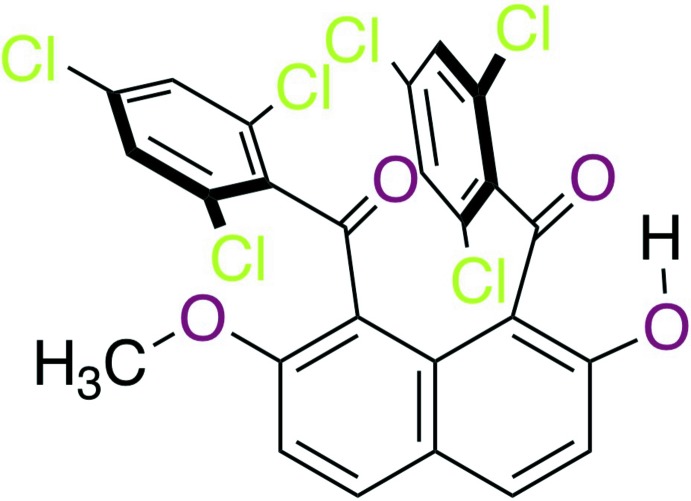


## Structural commentary   

**Figure 1:**
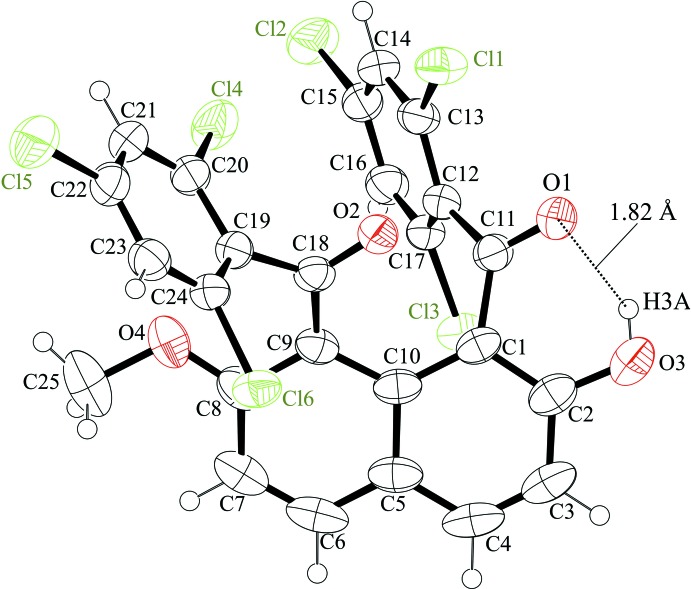
The mol­ecular structure of the title compound, with atom labelling. Displacement ellipsoids are drawn at the 50% probability level.

The mol­ecular structure of the title compound is shown in Fig. 1 ▸. This compound consists of a naphthalene ring core with two 2,4,6-tri­chloro­benzoyl groups at the 1,8-positions, a hy­droxy group at the 2-position, and a meth­oxy group at the 7-position of the naphthalene ring system, affording an unsymmetrical mol­ecular structure.

**Figure 2:**
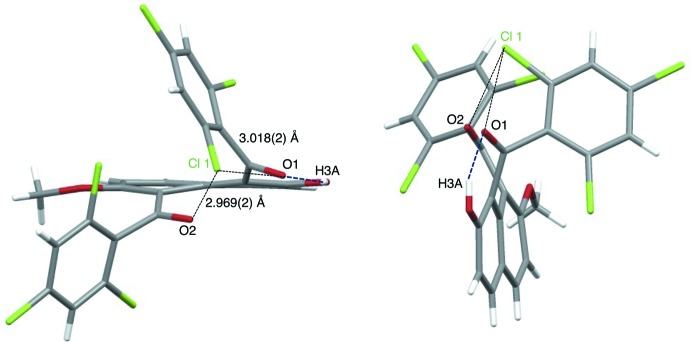
View of the title compound showing the intra­molecular contacts; top view (left) and side view (right).

Analogous aroylated unsymmetrical naphthalene compounds, for example, 1,8-bis­(*4-chloro*benzo­yl)-2-hy­droxy-7-meth­oxy­naphthalene (Mitsui, Nagasawa, Noguchi *et al.*, 2010 ▸) and 1,8-bis­(*4-chloro*benzo­yl)-2,7-*dimeth­oxy*naphthalene (Nakaema *et al.*, 2007 ▸), have two aroyl groups at the 1,8-positions of the naphthalene ring system. The two 4-chloro­benzoyl groups have the same orientation with respect to the naphthalene ring core in 1,8-bis­(*4-chloro*benzo­yl)-2-hy­droxy-7-meth­oxy­naphthalene, while they are in opposite directions in 1,8-bis­(*4-chloro*benzo­yl)-2,7-*dimeth­oxy*naphthalene. In contrast, in the title compound the carbonyl groups and the benzene rings of the 2,4,6-tri­chloro­benzoyl groups are located in distinct orientations with respect to the naphthalene ring plane: the two carbonyl groups are oriented in the same direction and are located roughly parallel to the naphthalene ring, whereas the two 2,4,6-tri­chloro­benzene rings are twisted away in opposite directions (Fig. 2 ▸). The dihedral angles of the carbonyl C—(C=O)—C plane [C1—(C11=O1)—C12 and C9—(C18=O2)—C19] and the naphthalene ring are 45.54 (15) and 30.02 (15)°, respectively. The carbonyl C—(C=O)—C plane and the 2,4,6-tri­chloro­benzene ring in the 8-position of the naphthalene ring forms a larger dihedral angle than that in 1-position [C19–C24 ring and C12–C17 ring], 79.78 (16)° *versus* 46.39 (16)°. The two carbonyl C—(C=O)—C planes make a large dihedral angle, 73.68 (19)°. Furthermore, the naphthalene ring plane is somewhat distorted, the C6—C5—C10—C9 and C4—C5—C10—C1 torsion angles being 10.2 (4) and 6.1 (5)°, respectively.

The intra­molecular O—H⋯O=C hydrogen bond forms a six-membered *S*(6) ring motif (O3—H3*A*⋯O1; Figs. 1 ▸ and 2 ▸, Table 1 ▸). In addition, one chloro atom of the tri­chloro­benzoyl group at the 1-position of the naphthalene ring system makes two short intra­molecular Cl⋯O=C contacts [Cl1⋯O1 = 3.018 (2) Å and Cl1⋯O2 = 2.969 (2) Å]. 1-Aroyl-2-hy­droxy­naphthalene homologues often form intra­molecular O—H⋯O=C hydrogen bonds whether the second aroyl group is present or not, *e.g.*, 1-benzoyl-2-hy­droxy-7-meth­oxy­naph­thalene (Nagasawa, Mitsui, Kato *et al.*, 2010 ▸), 2-hy­droxy-7-­meth­oxy-1-(4-methyl­benzo­yl)­naphthalene (Nagasawa, Mitsui, Okamoto *et al.*, 2010 ▸), 1-(4-chloro­benzo­yl)-2-hy­droxy-7-­meth­oxynaphthalene (Mitsui *et al.*, 2008 ▸) and 1,8-bis­(*4-chloro*benzo­yl)-2-hy­droxy-7-meth­oxy­naphthalene (Mitsui, Nagasawa, Noguchi *et al.*, 2010 ▸). The 2,4,6-trisubstituents in the benzene ring tend to bring about intra­molecular short contacts involving the carbonyl oxygen atom: intra­molecular C—H⋯O=C hydrogen bonds are observed in 2,7-dimeth­oxy-1,8-bis­(2,4,6-tri­methyl­benzo­yl)naphthalene (Muto *et al.*, 2012*a* ▸) and 1-(4-chloro­benzo­yl)-2,7-dimeth­oxy-8-(2,4,6-tri­methyl­benzo­yl)naphthalene (Muto *et al.*, 2012*b* ▸).

## Supra­molecular features   

**Figure 3:**
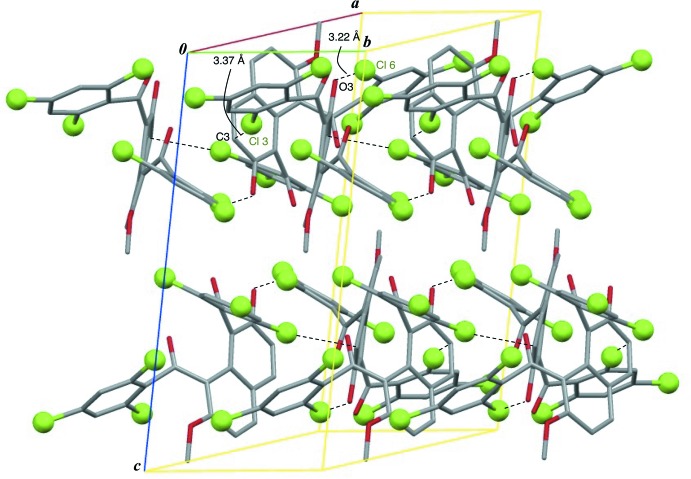
A view of the crystal packing of the title compound, showing the Cl⋯O and Cl⋯C short contacts. H atoms have been omitted for clarity.

In the crystal, 2_1_ helical mol­ecular assemblies are observed along the *b-*axis direction (Fig. 3 ▸). The chloro groups in the assemblies are aligned in a herringbone pattern. There are no effective hydrogen bonds, instead, two kinds of short contacts involving chlorine atoms are observed; Cl6⋯O3^i^ [3.224 (3) Å] and Cl3⋯C3^i^ [3.370 (3) Å], symmetry code: (i) −*x* + 1, *y* + 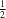, 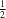 − *z* (Fig. 3 ▸).

## Hirshfeld surface analysis and two-dimensional fingerprint plots   

**Figure 4:**
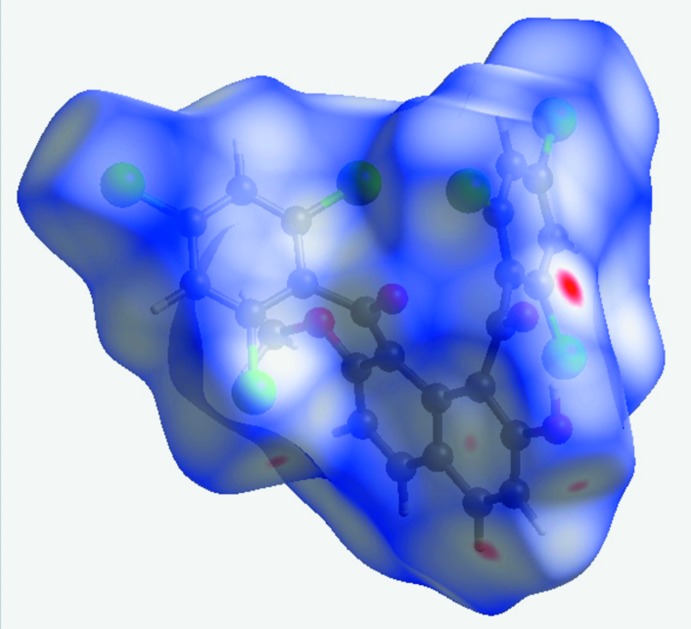
The Hirshfeld surface of the title compound mapped over *d*_norm_, in the range −0.0895 to 1.1549 a.u.

The Hirshfeld surface analysis (Spackman & Jayatilaka, 2009 ▸) and the associated two-dimensional fingerprint plots (McKinnon *et al.*, 2007 ▸) were performed with *CrystalExplorer17* (Turner *et al.*, 2017 ▸). The Hirshfeld surfaces are colour-mapped with the normalized contact distance, *d*_norm_, from red (distances shorter than the sum of the van der Waals radii) through white to blue (distances longer than the sum of the van der Waals radii). The Hirshfeld surface of the title compound mapped over *d*_norm_ in the range −0.0895 to 1.1549 a.u., is shown in Fig. 4 ▸. The red points represent close contacts and negative *d*_norm_ values on the surface. The largest red point corresponds to the short contact of 3.078 (3) Å involving the carbonyl O atom, O1, and carbon atom C23^i^ [symmetry code: (i) *x*, −*y* + 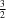, z − 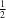], while the other red points around the naphthalene ring indicate short Cl⋯H inter­actions.

**Figure 5:**
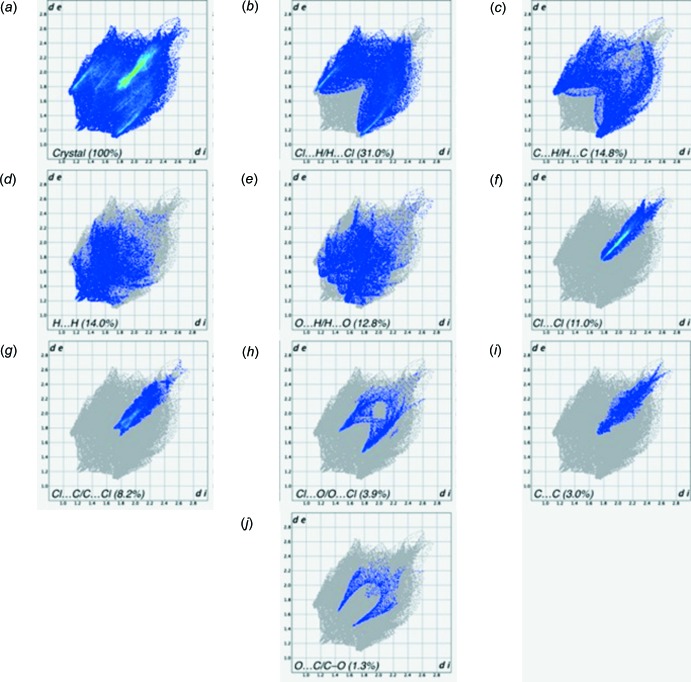
(*a*) The full two-dimensional fingerprint plot for the title compound, and those delineated into (*b*) Cl⋯H/H⋯Cl, (*c*) C⋯H/H⋯C, (*d*) H⋯H, (*e*) O⋯H/H⋯O, (*f*) Cl⋯Cl, (*g*) Cl⋯C, (*h*) Cl⋯O, (i) C⋯C and (*j*) O⋯C contacts.

The two-dimensional fingerprint plots from the Hirshfeld surface analysis are shown in Fig. 5 ▸, revealing the inter­molecular contacts and their percentage distributions on the Hirshfeld surface. Not surprisingly the Cl⋯H/H⋯Cl contacts (31.0%) are present as a major contributor, while C⋯H/H⋯C (14.8%), H⋯H (14.0%), O⋯H/H⋯O (12.8%), Cl⋯Cl (11.0%), Cl⋯C/C⋯Cl (8.2%), Cl⋯O/O⋯Cl (3.9%), C⋯C (3.0%) and O⋯C/C⋯O (1.3%) contacts also make significant contributions to the Hirshfeld surface.

## Database survey   

A search of the Cambridge Structural Database (CSD, version 5.40, last update November 2018; Groom *et al.*, 2016 ▸) of the 2-hy­droxy-1-benzoyl­naphthalene moiety of the title compound yield 16 hits. These include compounds with a similar aroyl­naphthalene unit and other polycyclic aromatic hydro­carbon moieties (CSD refcode ITUXOM: Ji *et al.*, 2016 ▸; PIRLUX: Freeman *et al.*, 1994 ▸; VUDFAC: Luo & Yu, 2009 ▸; VUDFEG: Luo & Yu, 2009 ▸). A search with a 1-benzoyl group bonded to the 2-hy­droxy­naphthalene framework gave 12 hits. Among these, three had bromo group(s) at the 8-position, 3,8-positions, and 3,6-positions of the naphthalene ring core, *viz.* PUKGIM (Mitsui, Nakaema, Nagasawa *et al.*, 2010 ▸), YUNWOP (Mitsui, Watanabe *et al.*, 2010 ▸) and YUPWEM (Mitsui, Nagasawa, Watanabe *et al.*, 2010 ▸). Four compounds had an 8-benzoyl group, *i.e.*, 1,8-diaroylated naphthalene compounds, *viz*. CIQBUB (Mohri *et al.*, 2013 ▸), LESLOM (Hijikata *et al.*, 2013 ▸), YUQBOC (Mitsui, Nagasawa, Noguchi *et al.*, 2010 ▸) and YUQBOC1 (Okamoto, Mitsui *et al.*, 2012 ▸). The remaining five compounds have a single 1-benzoyl-2-hy­droxy­naphthalene moiety, *viz*. KABJUU (Nagasawa, Mitsui, Kato *et al.*, 2010 ▸), UCUHAE (Okamoto *et al.*, 2014 ▸), VABBEH (Nagasawa, Mitsui, Okamoto *et al.*, 2010 ▸), VOJFOQ (Mitsui *et al.*, 2008 ▸) and VOJFQ01 (Okamoto, Mitsui *et al.*, 2012 ▸). These structures have *p*-substituted or unsubstituted benzoyl group(s). The structure most similar to the title compound is 1,8-bis­(4-chloro­benzo­yl)-7-meth­oxy­naphthalen-2-ol ethanol solvate, for which there are two reports; refcodes YUQBOC and YUQBOC01.

## Synthesis and crystallization   

To a 10 ml eggplant flask equipped with a nitro­gen bulb, 2,4,6-tri­chloro­benzoyl chloride (0.0938 ml, 0.6 mmol), di­chloro­methane (0.5 ml), titanium tetra­chloride (0.1972 ml, 1.8 mmol), and finally 2,7-di­meth­oxy­naphthalene (37.6 mg, 0.2 mmol) were introduced sequentially. The reaction mixture was stirred at ambient temperature for 6 h, then it was poured into ice–water. The resulting mixture was extracted with chloro­form (3 × 20 ml), then the organic layer was washed with saturated aqueous NaCl solution (3 × 20 ml) and dried over granular MgSO_4_. The solvent was removed by evaporation to yield a crude product of purple viscous liquid, which was crystallized from hot (hexa­ne/CHCl_3_) to give yellow plate-like crystals (isolated yield 24%; m.p. 493–497 K).

^1^H NMR δ (300 MHz, DMSO-*d*_6_); 3.59 (3H, s), 7.01 (1H, *d*, *J* = 8.4 Hz), 7.34 (1H, *d*, *J* = 9.3 Hz), 7.66 (2*H*, *s*), 7.70 (2H, *s*), 8.04 (1H, *d*, *J* = 8.7 Hz), 8.09 (1H, *d*, *J* = 9.0 Hz) ppm.

^1^H NMR δ (300 MHz, CDCl_3_); 3.45 (3H, *s*), 6.95 (1H, *br*), 7.02 (1H, *d*, *J* = 8.7 Hz), 7.20 (2H, *br*), 7.21 (1*H*, d, *J* = 9.0 Hz), 7.42 (1H, *br*), 7.90 (1H, *d*, *J* = 8.4 Hz), 8.01 (1H, *d*, *J* = 9.0 Hz) ppm.

^13^C NMR δ (100 MHz, CDCl_3_); 56.64, 110.35, 114.82, 117.67, 119.13, 119.38, 124.45, 127.37, 133.42, 133.68, 134.14, 135.42, 135.51, 136.69, 136.80, 139.15, 140.88, 165.60, 166.21, 185.98, 191.21 ppm.

IR (KBr); 1629 (C=O), 1600, 1510, 1442 (Ar, naphthalene), 1289 (=C—O—C) cm^−1^.

## Refinement   

Crystal data, data collection and structure refinement details are summarized in Table 2 ▸. All of the H atoms were found in a difference-Fourier map and were subsequently refined as riding atoms, with C—H = 0.95 (aromatic) and 0.96 (meth­yl) Å, and with *U*_iso_(H) = 1.2*U*_eq_(C).

## Supplementary Material

Crystal structure: contains datablock(s) I, Global. DOI: 10.1107/S2056989019012118/su5512sup1.cif

Structure factors: contains datablock(s) I. DOI: 10.1107/S2056989019012118/su5512Isup2.hkl

Supporting information file. DOI: 10.1107/S2056989019012118/su5512Isup3.cdx

1H NMR spectrum (in CDCl3, from -0.5 ppm to 12.5 ppm). DOI: 10.1107/S2056989019012118/su5512sup4.pdf

1H NMR spectrum (in CDCl3, from 6.5 ppm to 8.5 ppm). DOI: 10.1107/S2056989019012118/su5512sup5.pdf

13C NMR spectrum (in CDCl3). DOI: 10.1107/S2056989019012118/su5512sup6.pdf

IR spectrum. DOI: 10.1107/S2056989019012118/su5512sup7.pdf

CCDC reference: 1950725

Additional supporting information:  crystallographic information; 3D view; checkCIF report

## Figures and Tables

**Table 1:** Hydrogen-bond geometry (Å, °)

*D*—H⋯*A*	*D*—H	H⋯*A*	*D*⋯*A*	*D*—H⋯*A*
O3—H3*A*⋯O1	0.84	1.82	2.551 (3)	145

**Table 2:** Experimental details

Crystal data	
Chemical formula	C_25_H_12_Cl_6_O_4_
*M*_r_	589.05
Crystal system, space group	Monoclinic, *P*2_1_/*c*
Temperature (K)	193
*a*, *b*, *c* (Å)	17.9667 (4), 7.9150 (1), 17.6995 (5)
β (°)	110.673 (1)
*V* (Å^3^)	2354.91 (9)
*Z*	4
Radiation type	Cu *K*α
μ (mm^−1^)	6.95
Crystal size (mm)	0.40 × 0.40 × 0.20

Data collection	
Diffractometer	Rigaku R-AXIS RAPID
Absorption correction	Numerical (*NUMABS*; Higashi, 1999 ▸)
*T*_min_, *T*_max_	0.168, 0.337
No. of measured, independent and observed [*I* > 2σ(*I*)] reflections	41619, 4300, 3737
*R*_int_	0.110
(sin θ/λ)_max_ (Å^−1^)	0.602

Refinement	
*R*[*F* ^2^ > 2σ(*F* ^2^)], *wR*(*F* ^2^), *S*	0.045, 0.126, 1.06
No. of reflections	4300
No. of parameters	319
H-atom treatment	H-atom parameters constrained
Δρ_max_, Δρ_min_ (e Å^−3^)	0.50, −0.26

Computer programs: *PROCESS-AUTO* (Rigaku, 1998 ▸), *SIR2004* (Burla *et al.*, 2007 ▸), *SHELXL97* (Sheldrick, 2008 ▸) and *ORTEPIII* (Burnett & Johnson, 1996 ▸).

## supplementary crystallographic information

### Crystal data

**Table:**

C_25_H_12_Cl_6_O_4_	*F*(000) = 1184
*M**_r_* = 589.05	*D*_x_ = 1.661 Mg m^−^^3^
Monoclinic, *P*2_1_/*c*	Cu *K*α radiation, λ = 1.54187 Å
Hall symbol: -P 2ybc	Cell parameters from 28116 reflections
*a* = 17.9667 (4) Å	θ = 3.0–68.3°
*b* = 7.9150 (1) Å	µ = 6.95 mm^−^^1^
*c* = 17.6995 (5) Å	*T* = 193 K
β = 110.673 (1)°	Platelet, yellow
*V* = 2354.91 (9) Å^3^	0.40 × 0.40 × 0.20 mm
*Z* = 4	

### Data collection

**Table:**

Rigaku R-AXIS RAPID diffractometer	4300 independent reflections
Radiation source: rotaing anode	3737 reflections with *I* > 2σ(*I*)
Graphite monochromator	*R*_int_ = 0.110
Detector resolution: 10.000 pixels mm^-1^	θ_max_ = 68.3°, θ_min_ = 5.1°
ω scans	*h* = −21→21
Absorption correction: numerical (NUMABS; Higashi, 1999)	*k* = −9→9
*T*_min_ = 0.168, *T*_max_ = 0.337	*l* = −21→21
41619 measured reflections	

### Refinement

**Table:**

Refinement on *F*^2^	Secondary atom site location: difference Fourier map
Least-squares matrix: full	Hydrogen site location: inferred from neighbouring sites
*R*[*F*^2^ > 2σ(*F*^2^)] = 0.045	H-atom parameters constrained
*wR*(*F*^2^) = 0.126	*w* = 1/[σ^2^(*F*_o_^2^) + (0.0625*P*)^2^ + 1.5934*P*] where *P* = (*F*_o_^2^ + 2*F*_c_^2^)/3
*S* = 1.06	(Δ/σ)_max_ < 0.001
4300 reflections	Δρ_max_ = 0.50 e Å^−^^3^
319 parameters	Δρ_min_ = −0.26 e Å^−^^3^
0 restraints	Extinction correction: SHELXL97 (Sheldrick, 2008), Fc^*^=kFc[1+0.001xFc^2^λ^3^/sin(2θ)]^-1/4^
Primary atom site location: structure-invariant direct methods	Extinction coefficient: 0.00166 (17)

### Special details

**Table:**

Geometry. All esds (except the esd in the dihedral angle between two l.s. planes) are estimated using the full covariance matrix. The cell esds are taken into account individually in the estimation of esds in distances, angles and torsion angles; correlations between esds in cell parameters are only used when they are defined by crystal symmetry. An approximate (isotropic) treatment of cell esds is used for estimating esds involving l.s. planes.
Refinement. Refinement of F^2^ against ALL reflections. The weighted R-factor wR and goodness of fit S are based on F^2^, conventional R-factors R are based on F, with F set to zero for negative F^2^. The threshold expression of F^2^ > 2sigma(F^2^) is used only for calculating R-factors(gt) etc. and is not relevant to the choice of reflections for refinement. R-factors based on F^2^ are statistically about twice as large as those based on F, and R- factors based on ALL data will be even larger.

### Fractional atomic coordinates and isotropic or equivalent isotropic displacement parameters (Å^2^)

**Table:**

	*x*	*y*	*z*	*U*_iso_*/*U*_eq_	
Cl1	0.13592 (4)	0.62787 (10)	0.05084 (5)	0.0543 (2)	
Cl2	0.00708 (5)	0.06020 (13)	0.10904 (5)	0.0645 (3)	
Cl3	0.32240 (4)	0.09099 (9)	0.20159 (4)	0.0477 (2)	
Cl4	0.09824 (5)	0.59970 (12)	0.24340 (5)	0.0634 (3)	
Cl5	0.10530 (6)	1.19664 (13)	0.39062 (6)	0.0703 (3)	
Cl6	0.37761 (4)	0.90853 (10)	0.39693 (4)	0.0512 (2)	
O1	0.30235 (12)	0.5154 (3)	0.07105 (12)	0.0517 (5)	
O2	0.26606 (11)	0.6928 (3)	0.20713 (11)	0.0422 (5)	
O3	0.44648 (13)	0.4173 (3)	0.11247 (14)	0.0589 (6)	
H3A	0.4035	0.4583	0.0812	0.071*	
O4	0.26290 (15)	0.5184 (3)	0.41656 (12)	0.0613 (6)	
C1	0.37736 (16)	0.4494 (4)	0.20717 (17)	0.0413 (6)	
C2	0.44482 (17)	0.4097 (4)	0.1885 (2)	0.0492 (7)	
C3	0.51384 (17)	0.3460 (4)	0.2480 (2)	0.0571 (9)	
H3	0.5593	0.3195	0.2347	0.069*	
C4	0.51548 (18)	0.3226 (4)	0.3237 (2)	0.0584 (9)	
H4	0.5612	0.2728	0.3625	0.070*	
C5	0.45068 (18)	0.3702 (4)	0.34747 (19)	0.0509 (8)	
C6	0.4532 (2)	0.3417 (4)	0.42678 (19)	0.0604 (9)	
H6	0.4992	0.2905	0.4644	0.072*	
C7	0.3920 (2)	0.3850 (4)	0.45164 (19)	0.0583 (9)	
H7	0.3938	0.3581	0.5046	0.070*	
C8	0.3262 (2)	0.4701 (4)	0.39744 (17)	0.0511 (8)	
C9	0.32242 (16)	0.5103 (4)	0.31878 (16)	0.0409 (6)	
C10	0.38174 (16)	0.4428 (4)	0.28935 (17)	0.0418 (6)	
C11	0.30243 (16)	0.4589 (4)	0.13587 (16)	0.0403 (6)	
C12	0.22876 (15)	0.3656 (4)	0.13489 (15)	0.0379 (6)	
C13	0.15151 (17)	0.4292 (4)	0.09543 (16)	0.0431 (7)	
C14	0.08428 (16)	0.3378 (4)	0.08818 (16)	0.0464 (7)	
H14	0.0331	0.3860	0.0625	0.056*	
C15	0.09164 (16)	0.1760 (4)	0.11843 (17)	0.0458 (7)	
C16	0.16576 (17)	0.1018 (4)	0.15470 (16)	0.0457 (7)	
H16	0.1706	−0.0115	0.1736	0.055*	
C17	0.23220 (15)	0.1976 (4)	0.16237 (15)	0.0386 (6)	
C18	0.27267 (15)	0.6515 (4)	0.27587 (16)	0.0395 (6)	
C19	0.23318 (16)	0.7726 (4)	0.31692 (15)	0.0398 (6)	
C20	0.15062 (16)	0.7704 (4)	0.29719 (16)	0.0452 (7)	
C21	0.11036 (18)	0.8971 (4)	0.31971 (18)	0.0500 (7)	
H21	0.0543	0.8925	0.3057	0.060*	
C22	0.15427 (18)	1.0321 (4)	0.36357 (18)	0.0503 (7)	
C23	0.23617 (18)	1.0364 (4)	0.38688 (17)	0.0480 (7)	
H23	0.2656	1.1279	0.4180	0.058*	
C24	0.27431 (16)	0.9059 (4)	0.36436 (16)	0.0424 (6)	
C25	0.2684 (3)	0.5207 (6)	0.4997 (2)	0.0862 (14)	
H25A	0.2202	0.5722	0.5036	0.103*	
H25B	0.3150	0.5868	0.5317	0.103*	
H25C	0.2736	0.4048	0.5205	0.103*	

### Atomic displacement parameters (Å^2^)

**Table:**

	*U*^11^	*U*^22^	*U*^33^	*U*^12^	*U*^13^	*U*^23^
Cl1	0.0419 (4)	0.0540 (5)	0.0572 (4)	0.0039 (3)	0.0054 (3)	0.0116 (3)
Cl2	0.0421 (4)	0.0769 (6)	0.0739 (5)	−0.0167 (4)	0.0198 (4)	0.0057 (4)
Cl3	0.0375 (4)	0.0437 (4)	0.0543 (4)	0.0019 (3)	0.0067 (3)	0.0027 (3)
Cl4	0.0434 (4)	0.0766 (6)	0.0739 (5)	−0.0202 (4)	0.0255 (4)	−0.0219 (4)
Cl5	0.0680 (6)	0.0673 (6)	0.0806 (6)	0.0184 (5)	0.0324 (5)	−0.0061 (4)
Cl6	0.0357 (4)	0.0568 (5)	0.0525 (4)	−0.0032 (3)	0.0049 (3)	−0.0037 (3)
O1	0.0434 (11)	0.0682 (15)	0.0466 (11)	−0.0015 (10)	0.0198 (9)	0.0071 (10)
O2	0.0418 (10)	0.0444 (11)	0.0401 (10)	0.0018 (9)	0.0139 (8)	0.0033 (8)
O3	0.0461 (12)	0.0644 (16)	0.0731 (15)	0.0009 (11)	0.0297 (11)	−0.0043 (12)
O4	0.0764 (16)	0.0679 (16)	0.0452 (12)	−0.0017 (13)	0.0284 (11)	0.0088 (11)
C1	0.0307 (13)	0.0372 (15)	0.0531 (16)	−0.0021 (11)	0.0111 (12)	−0.0008 (12)
C2	0.0356 (15)	0.0438 (18)	0.0666 (19)	−0.0069 (13)	0.0161 (14)	−0.0040 (14)
C3	0.0312 (15)	0.0483 (19)	0.086 (2)	−0.0005 (13)	0.0137 (15)	−0.0100 (17)
C4	0.0330 (15)	0.0430 (18)	0.079 (2)	0.0006 (13)	−0.0053 (15)	−0.0020 (16)
C5	0.0391 (16)	0.0430 (18)	0.0542 (17)	−0.0043 (13)	−0.0037 (13)	−0.0053 (13)
C6	0.057 (2)	0.0471 (19)	0.0503 (18)	−0.0023 (16)	−0.0145 (15)	0.0031 (14)
C7	0.069 (2)	0.052 (2)	0.0395 (16)	−0.0097 (17)	0.0013 (15)	0.0044 (14)
C8	0.0587 (19)	0.0451 (18)	0.0425 (15)	−0.0083 (15)	0.0093 (14)	0.0011 (13)
C9	0.0370 (14)	0.0414 (16)	0.0382 (14)	−0.0050 (12)	0.0057 (11)	0.0009 (12)
C10	0.0332 (14)	0.0337 (15)	0.0497 (15)	−0.0041 (11)	0.0036 (11)	0.0001 (12)
C11	0.0361 (14)	0.0421 (16)	0.0414 (14)	0.0020 (12)	0.0123 (11)	−0.0003 (12)
C12	0.0336 (13)	0.0445 (16)	0.0332 (13)	−0.0010 (12)	0.0090 (10)	−0.0010 (11)
C13	0.0384 (14)	0.0486 (17)	0.0371 (14)	0.0000 (13)	0.0068 (11)	0.0021 (12)
C14	0.0324 (14)	0.061 (2)	0.0421 (15)	0.0021 (13)	0.0085 (11)	−0.0004 (13)
C15	0.0350 (14)	0.059 (2)	0.0423 (15)	−0.0096 (13)	0.0124 (11)	−0.0020 (13)
C16	0.0418 (16)	0.0502 (19)	0.0429 (15)	−0.0053 (13)	0.0121 (12)	0.0032 (13)
C17	0.0329 (13)	0.0451 (16)	0.0351 (13)	0.0006 (12)	0.0088 (10)	−0.0021 (11)
C18	0.0312 (13)	0.0440 (16)	0.0398 (14)	−0.0047 (12)	0.0080 (11)	0.0000 (12)
C19	0.0358 (13)	0.0473 (17)	0.0367 (13)	−0.0022 (12)	0.0132 (11)	0.0027 (12)
C20	0.0366 (14)	0.0576 (19)	0.0413 (14)	−0.0087 (13)	0.0138 (11)	−0.0023 (13)
C21	0.0383 (15)	0.067 (2)	0.0480 (16)	0.0024 (14)	0.0186 (13)	0.0005 (15)
C22	0.0510 (17)	0.055 (2)	0.0489 (16)	0.0111 (15)	0.0225 (14)	0.0034 (14)
C23	0.0494 (17)	0.0501 (18)	0.0421 (15)	0.0006 (14)	0.0132 (13)	−0.0025 (13)
C24	0.0339 (14)	0.0523 (18)	0.0383 (14)	−0.0010 (12)	0.0096 (11)	0.0015 (12)
C25	0.127 (4)	0.090 (3)	0.049 (2)	−0.016 (3)	0.040 (2)	−0.003 (2)

### Geometric parameters (Å, º)

**Table:**

Cl1—C13	1.737 (3)	C7—H7	0.9500
Cl2—C15	1.731 (3)	C8—C9	1.406 (4)
Cl3—C17	1.740 (3)	C9—C10	1.444 (4)
Cl4—C20	1.728 (3)	C9—C18	1.465 (4)
Cl5—C22	1.731 (3)	C11—C12	1.511 (4)
Cl6—C24	1.738 (3)	C12—C13	1.408 (4)
O1—C11	1.231 (3)	C12—C17	1.409 (4)
O2—C18	1.224 (3)	C13—C14	1.374 (4)
O3—C2	1.358 (4)	C14—C15	1.376 (5)
O3—H3A	0.8400	C14—H14	0.9500
O4—C8	1.351 (4)	C15—C16	1.388 (4)
O4—C25	1.441 (4)	C16—C17	1.380 (4)
C1—C2	1.400 (4)	C16—H16	0.9500
C1—C10	1.430 (4)	C18—C19	1.522 (4)
C1—C11	1.487 (4)	C19—C24	1.388 (4)
C2—C3	1.407 (4)	C19—C20	1.399 (4)
C3—C4	1.342 (5)	C20—C21	1.375 (4)
C3—H3	0.9500	C21—C22	1.391 (5)
C4—C5	1.421 (5)	C21—H21	0.9500
C4—H4	0.9500	C22—C23	1.381 (4)
C5—C6	1.407 (5)	C23—C24	1.374 (4)
C5—C10	1.422 (4)	C23—H23	0.9500
C6—C7	1.365 (5)	C25—H25A	0.9800
C6—H6	0.9500	C25—H25B	0.9800
C7—C8	1.403 (5)	C25—H25C	0.9800
			
C2—O3—H3A	109.5	C12—C13—Cl1	121.4 (2)
C8—O4—C25	120.0 (3)	C13—C14—C15	119.5 (3)
C2—C1—C10	119.4 (3)	C13—C14—H14	120.2
C2—C1—C11	114.3 (3)	C15—C14—H14	120.2
C10—C1—C11	125.0 (2)	C14—C15—C16	121.2 (3)
O3—C2—C1	123.2 (3)	C14—C15—Cl2	119.6 (2)
O3—C2—C3	115.8 (3)	C16—C15—Cl2	119.1 (3)
C1—C2—C3	120.8 (3)	C17—C16—C15	117.9 (3)
C4—C3—C2	120.0 (3)	C17—C16—H16	121.0
C4—C3—H3	120.0	C15—C16—H16	121.0
C2—C3—H3	120.0	C16—C17—C12	123.6 (3)
C3—C4—C5	121.8 (3)	C16—C17—Cl3	115.1 (2)
C3—C4—H4	119.1	C12—C17—Cl3	121.1 (2)
C5—C4—H4	119.1	O2—C18—C9	123.1 (3)
C6—C5—C4	120.7 (3)	O2—C18—C19	114.1 (2)
C6—C5—C10	120.0 (3)	C9—C18—C19	122.4 (2)
C4—C5—C10	119.2 (3)	C24—C19—C20	116.9 (3)
C7—C6—C5	122.4 (3)	C24—C19—C18	122.0 (2)
C7—C6—H6	118.8	C20—C19—C18	120.3 (2)
C5—C6—H6	118.8	C21—C20—C19	122.6 (3)
C6—C7—C8	118.7 (3)	C21—C20—Cl4	119.3 (2)
C6—C7—H7	120.7	C19—C20—Cl4	118.1 (2)
C8—C7—H7	120.7	C20—C21—C22	118.0 (3)
O4—C8—C7	123.0 (3)	C20—C21—H21	121.0
O4—C8—C9	115.7 (3)	C22—C21—H21	121.0
C7—C8—C9	121.3 (3)	C23—C22—C21	121.3 (3)
C8—C9—C10	119.3 (3)	C23—C22—Cl5	119.4 (3)
C8—C9—C18	119.6 (3)	C21—C22—Cl5	119.3 (2)
C10—C9—C18	119.2 (2)	C24—C23—C22	118.9 (3)
C5—C10—C1	118.1 (3)	C24—C23—H23	120.6
C5—C10—C9	117.0 (3)	C22—C23—H23	120.6
C1—C10—C9	124.8 (2)	C23—C24—C19	122.2 (3)
O1—C11—C1	120.8 (3)	C23—C24—Cl6	118.6 (2)
O1—C11—C12	117.0 (2)	C19—C24—Cl6	119.2 (2)
C1—C11—C12	120.8 (2)	O4—C25—H25A	109.5
C13—C12—C17	115.1 (3)	O4—C25—H25B	109.5
C13—C12—C11	122.5 (3)	H25A—C25—H25B	109.5
C17—C12—C11	121.7 (2)	O4—C25—H25C	109.5
C14—C13—C12	122.6 (3)	H25A—C25—H25C	109.5
C14—C13—Cl1	116.0 (2)	H25B—C25—H25C	109.5
			
C10—C1—C2—O3	−178.1 (3)	C17—C12—C13—C14	3.5 (4)
C11—C1—C2—O3	14.5 (4)	C11—C12—C13—C14	174.0 (3)
C10—C1—C2—C3	7.0 (4)	C17—C12—C13—Cl1	−175.0 (2)
C11—C1—C2—C3	−160.4 (3)	C11—C12—C13—Cl1	−4.6 (4)
O3—C2—C3—C4	−175.3 (3)	C12—C13—C14—C15	−1.8 (4)
C1—C2—C3—C4	0.0 (5)	Cl1—C13—C14—C15	176.8 (2)
C2—C3—C4—C5	−3.9 (5)	C13—C14—C15—C16	−1.4 (4)
C3—C4—C5—C6	178.9 (3)	C13—C14—C15—Cl2	−179.7 (2)
C3—C4—C5—C10	0.8 (5)	C14—C15—C16—C17	2.5 (4)
C4—C5—C6—C7	−179.7 (3)	Cl2—C15—C16—C17	−179.2 (2)
C10—C5—C6—C7	−1.6 (5)	C15—C16—C17—C12	−0.5 (4)
C5—C6—C7—C8	−3.9 (5)	C15—C16—C17—Cl3	−175.5 (2)
C25—O4—C8—C7	15.0 (5)	C13—C12—C17—C16	−2.3 (4)
C25—O4—C8—C9	−165.1 (3)	C11—C12—C17—C16	−172.9 (3)
C6—C7—C8—O4	−179.8 (3)	C13—C12—C17—Cl3	172.3 (2)
C6—C7—C8—C9	0.2 (5)	C11—C12—C17—Cl3	1.8 (4)
O4—C8—C9—C10	−171.3 (3)	C8—C9—C18—O2	−179.4 (3)
C7—C8—C9—C10	8.7 (4)	C10—C9—C18—O2	16.3 (4)
O4—C8—C9—C18	24.5 (4)	C8—C9—C18—C19	8.5 (4)
C7—C8—C9—C18	−155.6 (3)	C10—C9—C18—C19	−155.8 (3)
C6—C5—C10—C1	−172.0 (3)	O2—C18—C19—C24	−91.9 (3)
C4—C5—C10—C1	6.1 (4)	C9—C18—C19—C24	80.8 (4)
C6—C5—C10—C9	10.2 (4)	O2—C18—C19—C20	77.3 (3)
C4—C5—C10—C9	−171.7 (3)	C9—C18—C19—C20	−109.9 (3)
C2—C1—C10—C5	−9.9 (4)	C24—C19—C20—C21	3.1 (4)
C11—C1—C10—C5	156.1 (3)	C18—C19—C20—C21	−166.7 (3)
C2—C1—C10—C9	167.7 (3)	C24—C19—C20—Cl4	−176.2 (2)
C11—C1—C10—C9	−26.3 (4)	C18—C19—C20—Cl4	14.1 (4)
C8—C9—C10—C5	−13.6 (4)	C19—C20—C21—C22	0.2 (4)
C18—C9—C10—C5	150.6 (3)	Cl4—C20—C21—C22	179.5 (2)
C8—C9—C10—C1	168.7 (3)	C20—C21—C22—C23	−2.6 (4)
C18—C9—C10—C1	−27.0 (4)	C20—C21—C22—Cl5	178.3 (2)
C2—C1—C11—O1	−36.1 (4)	C21—C22—C23—C24	1.6 (4)
C10—C1—C11—O1	157.3 (3)	Cl5—C22—C23—C24	−179.3 (2)
C2—C1—C11—C12	130.2 (3)	C22—C23—C24—C19	2.0 (4)
C10—C1—C11—C12	−36.4 (4)	C22—C23—C24—Cl6	−177.8 (2)
O1—C11—C12—C13	−47.0 (4)	C20—C19—C24—C23	−4.2 (4)
C1—C11—C12—C13	146.3 (3)	C18—C19—C24—C23	165.4 (3)
O1—C11—C12—C17	122.9 (3)	C20—C19—C24—Cl6	175.5 (2)
C1—C11—C12—C17	−43.9 (4)	C18—C19—C24—Cl6	−14.9 (4)

### Hydrogen-bond geometry (Å, º)

**Table:**

*D*—H···*A*	*D*—H	H···*A*	*D*···*A*	*D*—H···*A*
O3—H3*A*···O1	0.84	1.82	2.551 (3)	145

## References

[bb1] Burla, M. C., Caliandro, R., Camalli, M., Carrozzini, B., Cascarano, G. L., De Caro, L., Giacovazzo, C., Polidori, G., Siliqi, D. & Spagna, R. (2007). *J. Appl. Cryst.* **40**, 609–613.

[bb2] Burnett, M. N. & &Johnson, C. K. (1996). *ORTEPIII*. Report ORNL-6895. Oak Ridge National Laboratory. Tennessee, USA.

[bb3] Choy, P. Y. & Kwong, F. Y. (2013). *Org. Lett.* **15**, 270–273. 10.1021/ol303088z 23301995

[bb4] Freeman, D., Frolow, F., Kapinus, E., Lavie, D., Lavie, G., Meruelo, D. & Mazur, Y. (1994). *J. Chem. Soc. Chem. Commun.* pp. 891–892.

[bb5] Groom, C. R., Bruno, I. J., Lightfoot, M. P. & Ward, S. C. (2016). *Acta Cryst.* B**72**, 171–179. 10.1107/S2052520616003954 PMC4822653 27048719

[bb6] Higashi, T. (1999). *NUMABS*. Rigaku Corporation, Tokyo, Japan.

[bb7] Hijikata, D., Sasagawa, K., Yoshiwaka, S., Okamoto, A. & Yonezawa, N. (2013). *Acta Cryst.* E**69**, o208–o209. 10.1107/S1600536812052038 PMC3569745 23424491

[bb8] Ji, K., Yang, F., Gao, S., Tang, J.-J. & Gao, J. (2016). *Chem. Eur. J.* **22**, 10225–10229. 10.1002/chem.201600736 27276524

[bb9] Lee, H. & Yi, C. S. (2015). *Eur. J. Org. Chem.* pp. 1899–1904. 10.1002/ejoc.201403518 PMC4493914 26167129

[bb10] Luo, N. & Yu, Z. (2009). *J. Organomet. Chem.* **694**, 3058–3067.

[bb11] McKinnon, J. J., Jayatilaka, D. & Spackman, M. A. (2007). *Chem. Commun.* pp. 3814–3816. 10.1039/b704980c 18217656

[bb12] Mitsui, R., Nagasawa, A., Noguchi, K., Okamoto, A. & Yonezawa, N. (2010). *Acta Cryst.* E**66**, o1790. 10.1107/S1600536810024074 PMC3007080 21588000

[bb13] Mitsui, R., Nagasawa, A., Watanabe, S., Okamoto, A. & Yonezawa, N. (2010). *Acta Cryst.* E**66**, o1761. 10.1107/S1600536810023299 PMC3007072 21587976

[bb14] Mitsui, R., Nakaema, K., Nagasawa, A., Noguchi, K. & Yonezawa, N. (2010). *Acta Cryst.* E**66**, o676. 10.1107/S1600536810006185 PMC2983574 21580422

[bb15] Mitsui, R., Nakaema, K., Noguchi, K. & Yonezawa, N. (2008). *Acta Cryst.* E**64**, o2497. 10.1107/S1600536808039603 PMC2959982 21581458

[bb16] Mitsui, R., Watanabe, S., Nagasawa, A., Okamoto, A. & Yonezawa, N. (2010). *Acta Cryst.* E**66**, o1304. 10.1107/S1600536810015527 PMC2979364 21579400

[bb17] Mohri, S., Yoshiwaka, S., Isozaki, K., Yonezawa, N. & Okamoto, A. (2013). *Acta Cryst.* C**69**, 1541–1544. 10.1107/S0108270113030771 24311508

[bb18] Murashige, R., Hayashi, Y., Ohmori, S., Torii, A., Aizu, Y., Muto, Y., Murai, Y., Oda, Y. & Hashimoto, M. (2011). *Tetrahedron*, **67**, 641–649.

[bb19] Muto, T., Sasagawa, K., Okamoto, A., Oike, H. & Yonezawa, N. (2012*a*). *Acta Cryst.* E**68**, o23. 10.1107/S1600536811051579 PMC3254363 22259511

[bb20] Muto, T., Sasagawa, K., Okamoto, A., Oike, H. & Yonezawa, N. (2012*b*). *Acta Cryst.* E**68**, o906. 10.1107/S1600536812008112 PMC3297949 22412752

[bb21] Naeimi, H., Amini, A. & Moradian, M. (2014). *Org. Chem. Front.* **1**, 415–421.

[bb22] Nagasawa, A., Mitsui, R., Kato, Y., Okamoto, A. & Yonezawa, N. (2010). *Acta Cryst.* E**66**, o2677. 10.1107/S1600536810038547 PMC2983170 21587645

[bb23] Nagasawa, A., Mitsui, R., Okamoto, A. & Yonezawa, N. (2010). *Acta Cryst.* E**66**, o2820–o2821. 10.1107/S1600536810040614 PMC3009359 21589009

[bb24] Nakaema, K., Okamoto, A., Noguchi, K. & Yonezawa, N. (2007). *Acta Cryst.* E**63**, o4120.

[bb25] Nimnual, P., Tummatorn, J., Thongsornkleeb, C. & Ruchirawat, S. (2015). *J. Org. Chem.* **80**, 8657–8667. 10.1021/acs.joc.5b01305 26244465

[bb26] Nonhebel, D. C. (1968). *Tetrahedron*, **24**, 1869–1874.

[bb27] Ohisa, S., Saeki, M., Shiomichi, H., Yonezawa, N. & Okamoto, A. (2018). *Eur. Chem. Bull.* **7**, 1–9.

[bb28] Okamoto, A., Mitsui, R., Oike, H. & Yonezawa, N. (2011). *Chem. Lett.* **40**, 1283–1284.

[bb29] Okamoto, A., Mitsui, R., Watanabe, S., Tsubouchi, T. & Yonezawa, N. (2012). *Int. J. Org. Chem.* **02**, 194–201.

[bb30] Okamoto, A., Nagasawa, A. & Yonezawa, N. (2014). *Eur. Chem. Bull.* **3**, 263–268.

[bb31] Okamoto, A., Watanabe, S., Nakaema, K. & Yonezawa, N. (2012). *Cryst. Struc. Theo. Appl.* **1**, 121–127.

[bb32] Okamoto, A. & Yonezawa, N. (2009). *Chem. Lett.* **38**, 914–915.

[bb33] Okamoto, A. & Yonezawa, N. (2015). *J. Syn. Org. Chem. Jpn.* **73**, 339–360.

[bb34] Rigaku (1998). *PROCESS-AUTO.* Rigaku Corporation, Tokyo, Japan.

[bb35] Schiel, C., Oelgemoller, M. & Mattay, J. (2001). *Synthesis*, pp. 1275–1279.

[bb36] Sheldrick, G. M. (2008). *Acta Cryst.* A**64**, 112–122. 10.1107/S0108767307043930 18156677

[bb37] Siskos, M. G., Tzakos, A. G. & Gerothanassis, I. P. (2015). *Org. Biomol. Chem.* **13**, 8852–8868. 10.1039/c5ob00920k 26196256

[bb38] Spackman, M. A. & Jayatilaka, D. (2009). *CrystEngComm*, **11**, 19–32.

[bb39] Tran, P. H., Phung, H. Q., Duong, M. N. & Pham-Tran, N.-N. (2017). *Tetrahedron Lett.* **58**, 1588–1563.

[bb40] Turner, M. J., McKinnon, J. J., Wolff, S. K., Grimwood, D. J., Spackman, P. R., Jayatilaka, D. & Spackman, M. A. (2017). *CrystalExplorer17. University of Western Australia.* http://hirshfeldsurface.net

[bb41] Weng, F., Wang, C. & Xu, B. (2010). *Tetrahedron Lett.* **51**, 2593–2596.

[bb42] Yoshiwaka, S., Ogata, K., Yonezawa, N. & Okamoto, A. (2015). *Eur. Chem. Bull.* **4**(4), 195–201.

[bb43] Zhou, C. & Larock, R. C. (2004). *J. Am. Chem. Soc.* **126**, 2302–2303. 10.1021/ja038687l 14982423

