# Supplementary material for: Crystal structure and Hirshfeld surface analysis of 2-hy­droxy-7-meth­oxy-1,8-bis­(2,4,6-tri­chloro­benzo­yl)naphthalene
Source: Acta Crystallogr E Crystallogr Commun. 2019 Sep 10;75(Pt 10):1418–22. doi: 10.1107/S2056989019012118 (PMC6775736; doi:10.1107/S2056989019012118)

single_pulse

C:\Documents and Settings\•ÄàVŒ¤‹†Ž°\My Documents\ŒÂlftfHf‹f_\Meg feat. Toyopon\NMR\M-2\H-1201\120123-1,8-triCl-2-OH-1.jdf

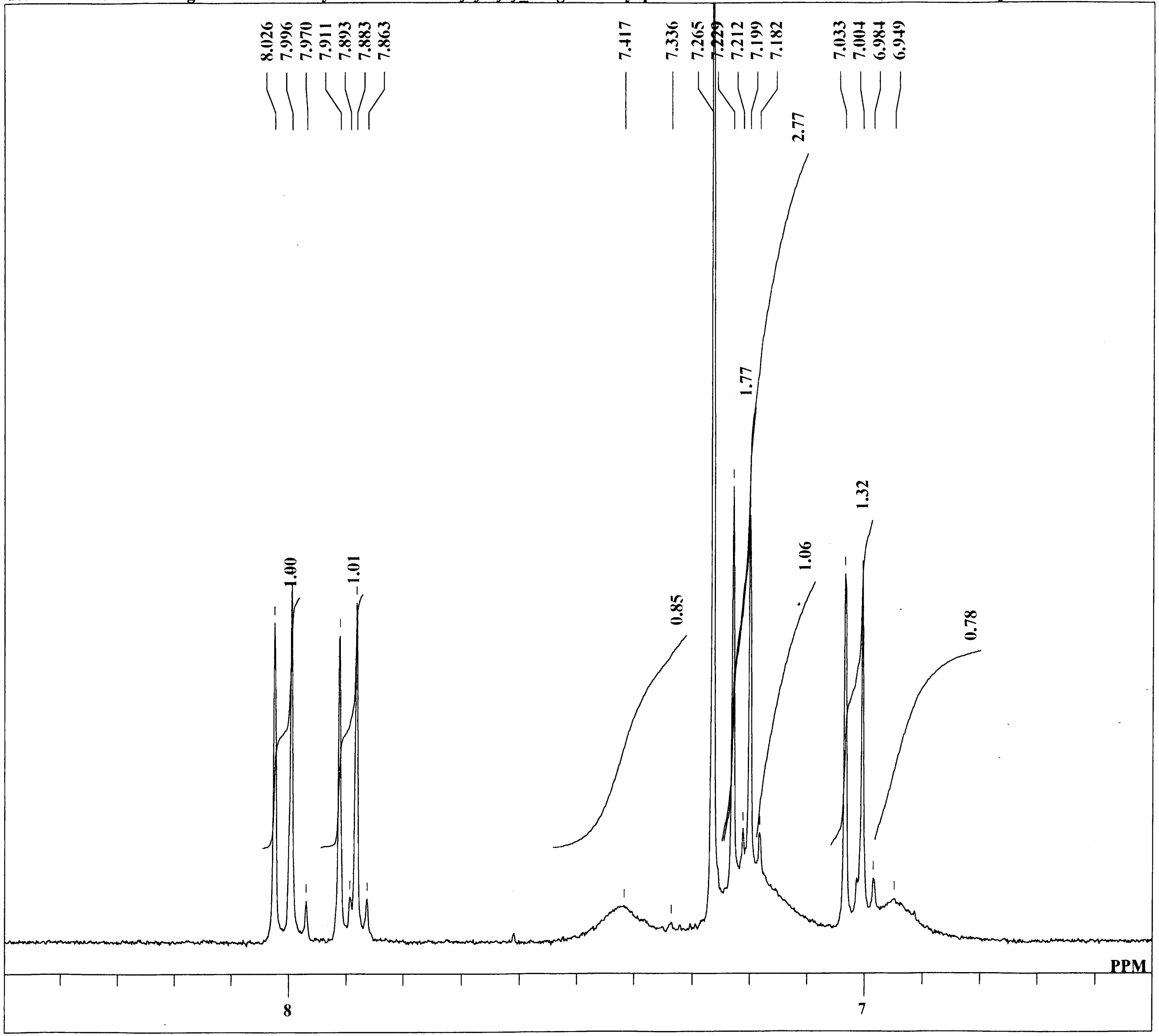

DFILE	120123-1,8-triCl-2-OH-1.jdf
COMNT	single_pulse
DATIM	23-01-2012 16:52:39
OBNUC	1H
EXMOD	single_pulse.ex2
OBFRQ	300.53 MHz
OBSET	1.15 KHz
OBFIN	8.57 Hz
POINT	16384
FREQU	5635.71 Hz
SCANS	4
ACQTM	2.9072 sec
PD	5.0000 sec
PW1	5.95 usec
IRNUC	1H
CTEMP	15.4 c
SLVNT	CDCL3
EXREF	0.00 ppm
BF	0.12 Hz
RGAIN	42

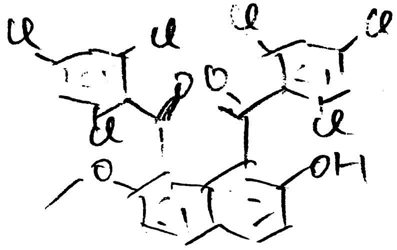

Supplement: Supplementary file 5 [file e-75-01418-sup5.pdf]
