# Supplementary material for: Crystal structure and Hirshfeld surface analysis of 2-hy­droxy-7-meth­oxy-1,8-bis­(2,4,6-tri­chloro­benzo­yl)naphthalene
Source: Acta Crystallogr E Crystallogr Commun. 2019 Sep 10;75(Pt 10):1418–22. doi: 10.1107/S2056989019012118 (PMC6775736; doi:10.1107/S2056989019012118)
Supplement: Supplementary file 6 [file e-75-01418-sup6.pdf]

single pulse decoupled gated NOE

H:¥NMR¥120228-1,8-triCl-2-OH-13C-1.jdf

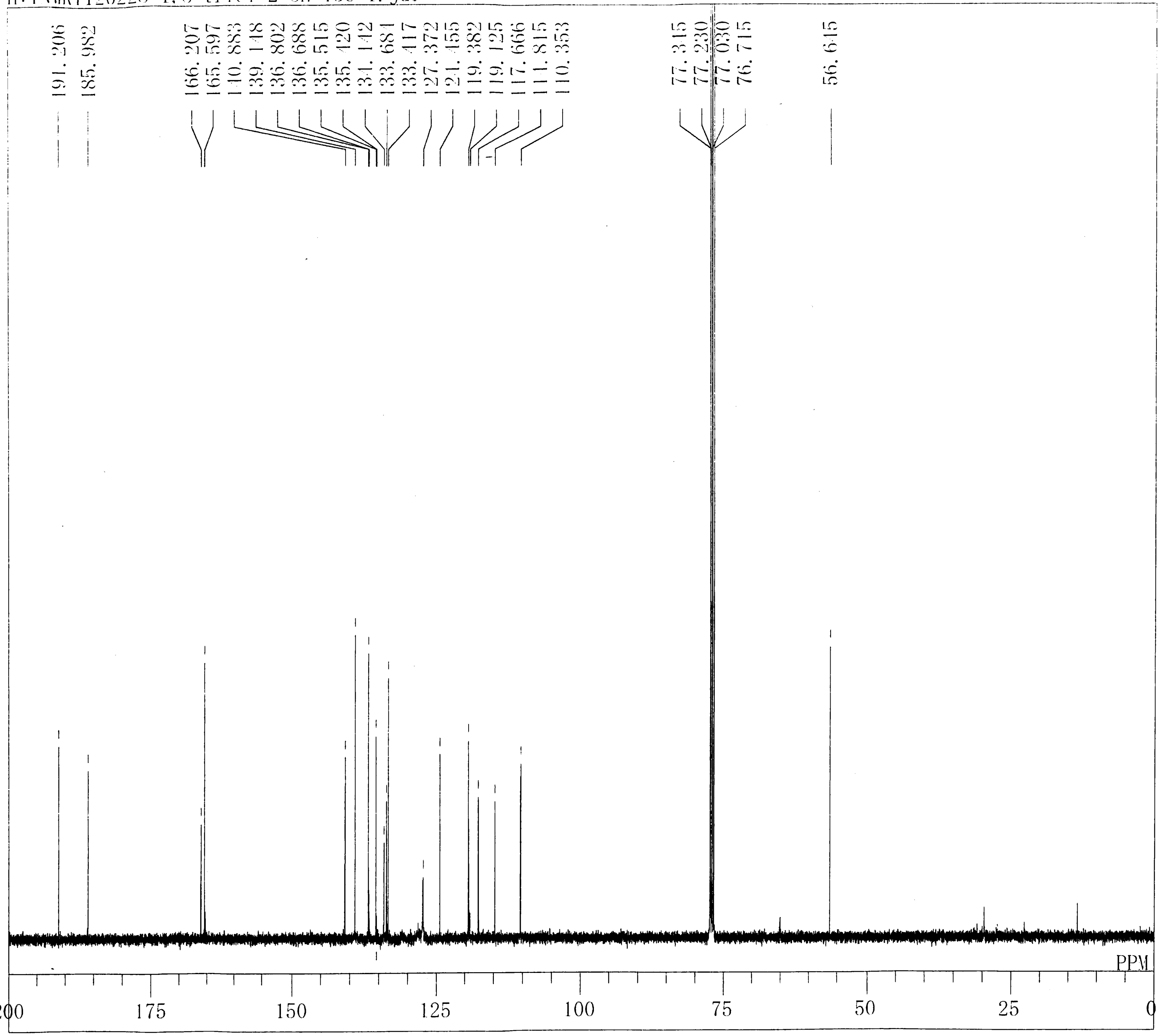


| | |
|---|---|
| DFILE | 120228-1,8-triCl-2-OH-13C-1. |
| COMNT | single pulse decoupled gated |
| DATIM | 28-02-2012 21:33:42 |
| OBNUC | 13C |
| EXMOD | single_pulse_dec |
| OBFRQ | 100.53 MHz |
| OBSET | 5.35 KHz |
| OBFIN | 5.86 Hz |
| POINT | 32768 |
| FREQU | 31407.03 Hz |
| SCANS | 3176 |
| ACQTM | 1.0433 sec |
| PD | 2.0000 sec |
| PW1 | 3.12 usec |
| IRNUC | 1H |
| CTEMP | 25.1 c |
| SLVNT | CDCL3 |
| EXREF | 0.00 ppm |
| BF | 0.12 Hz |
| RGAIN | 58 |
