# Supplementary material for: Crystal structure and Hirshfeld surface analysis of 2-hy­droxy-7-meth­oxy-1,8-bis­(2,4,6-tri­chloro­benzo­yl)naphthalene
Source: Acta Crystallogr E Crystallogr Commun. 2019 Sep 10;75(Pt 10):1418–22. doi: 10.1107/S2056989019012118 (PMC6775736; doi:10.1107/S2056989019012118)
Supplement: Supplementary file 7 [file e-75-01418-sup7.pdf]

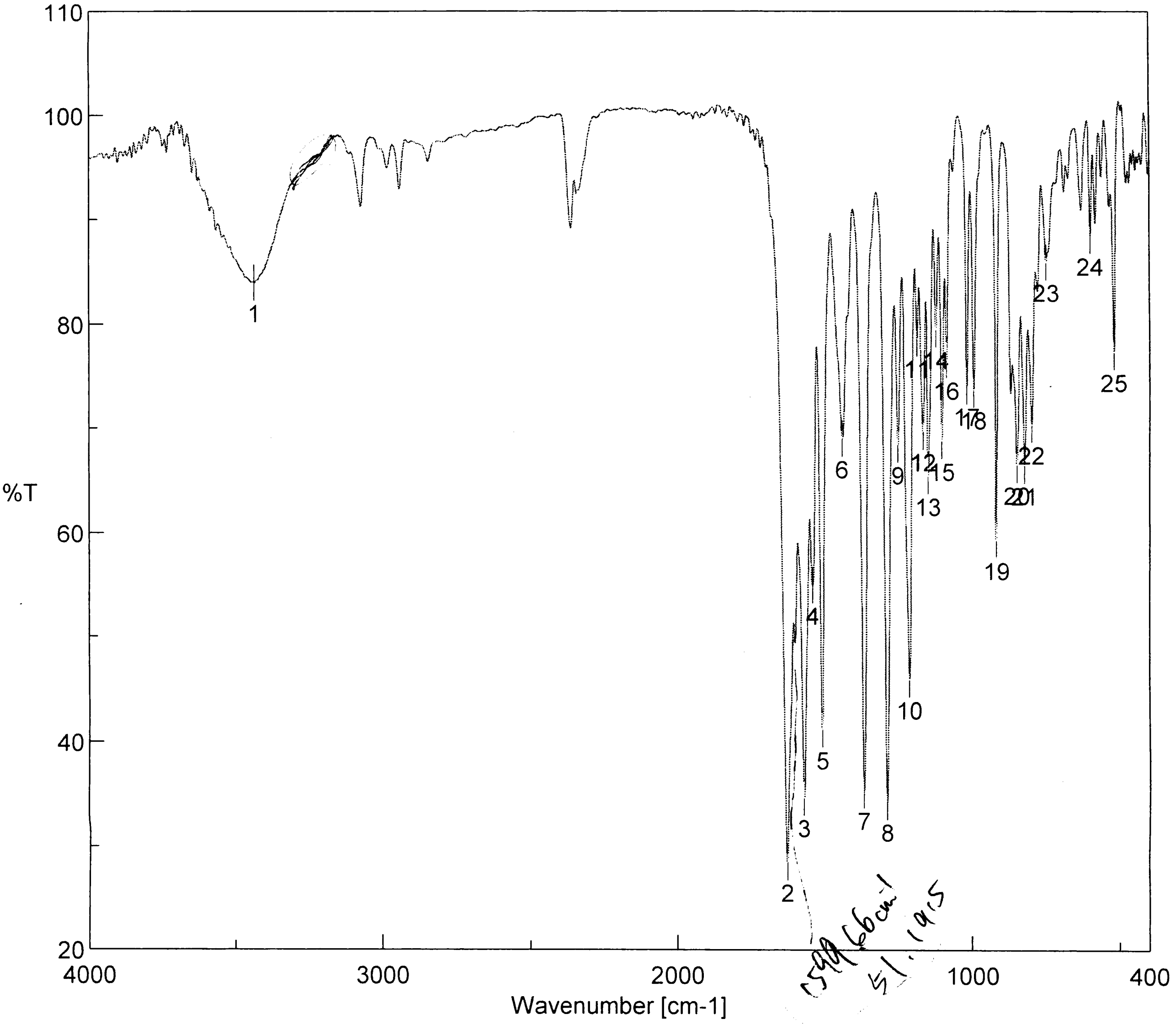

ピーク検出結果

No.	位置	強度	No.	位置	強度
1	3434.6	83.9172	2	1628.59	28.4918
3	1571.7	34.6546	4	1541.81	54.9725
5	1509.99	41.1432	6	1441.53	69.0207
7	1368.25	35.4156	8	1289.18	34.2781
9	1251.58	68.4691	10	1213.97	45.9814
11	1186.01	78.6403	12	1165.76	69.7077
13	1148.4	65.4522	14	1122.37	79.4709
15	1101.15	68.815	16	1085.73	76.5499
17	1016.3	74.0587	18	992.196	73.7066
19	916.022	59.3182	20	845.633	66.5387
21	819.598	66.5118	22	795.493	70.3913
23	746.317	85.99	24	596.861	88.5038
25	517.793	77.3523			

[コメント情報]

試料名	
コメント	
測定者	
所属	
会社	東京農工大学

[測定情報]

機種名	FT/IR-4100typeA
シリアル番号	B041461016
光源	標準光源
検出器	TGS
積算回数	32
分解	4 cm-1
ゼロフィリング	On
アポダイゼーション	Cosine
ゲイン	Auto (8)
アパーチャー	Auto (7.1 mm)
スキャンスピード	Auto (2 mm/sec)
フィルタ	Auto (30000 Hz)
